# Risk-Based Monitoring in Clinical Trials: Past, Present, and Future

**DOI:** 10.1007/s43441-021-00295-8

**Published:** 2021-04-29

**Authors:** Brian Barnes, Nicole Stansbury, Debby Brown, Lauren Garson, Geoff Gerard, Nickolas Piccoli, Debra Jendrasek, Nick May, Vanesa Castillo, Anina Adelfio, Nycole Ramirez, Andrea McSweeney, Ruth Berlien, Paula Jo Butler

**Affiliations:** Association of Clinical Research Organizations (ACRO), Washington, DC USA

## Abstract

Risk-based monitoring (RBM) is a powerful tool for efficiently ensuring patient safety and data integrity in a clinical trial, enhancing overall trial quality. To better understand the state of RBM implementation across the clinical trial industry, the Association of Clinical Research Organizations (ACRO) conducted a landscape survey among its member companies across 6,513 clinical trials ongoing at the end of 2019. Of these trials, 22% included at least 1 of the 5 RBM components: key risk indicators (KRIs), centralized monitoring, off-site/remote-site monitoring, reduced source data verification (SDV), and reduced source document review (SDR). The implementation rates for the individual RBM components ranged 8%–19%, with the most frequently implemented component being centralized monitoring and the least frequently implemented being reduced SDR. When the COVID-19 pandemic emerged in early 2020, additional data were collected to assess its impact on trial monitoring, focusing specifically on trials switching from on-site monitoring to off-site/remote-site monitoring. These mid-pandemic data show that the vast majority of monitoring visits were on-site in February 2020, but an even higher percentage were off-site in April, corresponding with the first peak of the pandemic. Despite this shift, similar numbers of non-COVID-related protocol deviations were detected from February through June, suggesting little or no reduction in monitoring effectiveness. The pre- and mid-pandemic data provide two very different snapshots of RBM implementation, but both support the need to promote adoption of this approach while also highlighting an opportunity to capitalize on the recent shift toward greater RBM uptake in a post-pandemic environment.

## Introduction

Clinical trial management is a complex endeavor requiring careful planning, compliance with regulations, and coordination between multiple stakeholders such as sponsors, investigators, and contract research organizations (CROs). Risk-based monitoring (RBM) of clinical trials has emerged as a more targeted, strategic approach that takes advantage of increased connectivity and advances in data analytics. RBM streamlines and optimizes error detection, which may facilitate replacement of some or all on-site monitoring visits. The aim of RBM is to focus monitoring on those trial processes most likely to affect patient safety and data quality, often using real-time analytics, so that investigators can more quickly and effectively mitigate risks or address errors before they compromise trial quality.

RBM is an important component of a larger framework known as risk-based quality management (RBQM), defined in a 2013 European Medicines Agency (EMA) reflection paper as “a systematic process put in place to identify, assess, control, communicate and review the risks associated with the clinical trial during its lifecycle” [1–3]. Compared with source data verification (SDV), source document review (SDR), and other forms of monitoring focused on past events, RBM has a stronger focus on the present and future, particularly when it includes real-time monitoring and predictive modeling [4]. These forward-looking activities impact not only monitoring functions but also overall trial management. In other words, RBQM is a holistic, quality management, systems-based approach to trial implementation, and RBM as a monitoring strategy is an integral part of that approach [2]. Importantly, RBQM also directly addresses the directives on RBM contained in the ICH E6(R2) guidance [5].

The key components of RBQM include the following:

1. Initial Cross-functional Risk Assessment—Involves multiple stakeholders and identifies critical-to-quality (critical data and critical process) risks across the entire trial lifecycle as well as mitigation strategies, which will inform project plans.
2. Ongoing Cross-functional Risk Assessment—A continuous process of revisiting and adjusting the initial risk assessment and planned mitigations as the trial proceeds based on incoming data and any new developments within or outside of the trial that could affect quality.
3. Quality Tolerance Limits (QTLs)—Pre-determined limits for specific trial parameters that, when reached, signal that further evaluation is needed to determine if action is warranted.
4. Key Risk Indicators (KRIs)—Metrics used to assess site performance, either compared to other sites or to established values.
5. Centralized Monitoring—The remote review of aggregated electronic data, including data analysis.
6. Off-Site/Remote-site Monitoring—Replacement of some or all on-site monitoring visits with remote-site monitoring visits, where and when allowed by regulatory authorities. When monitoring remotely, a targeted and/or triggered review of documents and data is used.
7. Reduced SDV—Shift from 100% SDV to more targeted monitoring.
8. Reduced SDR—Shift from 100% SDR to more targeted monitoring.

The definition of each component was agreed upon by the authors; however, different terminology may be used across the industry. Components 1–3 affect multiple trial activities beyond monitoring and thus help form the “backbone” of the holistic RBQM framework, while Components 4–8 comprise the monitoring activities and tools specific to RBM. Although the distinction between trial-level activities and monitoring is important, the critical risk assessment and QTL-setting functions of RBQM must also be implemented for RBM to be fully successful.

As a relatively mature concept, RBM offers established benefits to trial execution, including enhanced effectiveness of monitoring, increased overall trial quality, greater efficiency, improved patient safety, and better overall value [3, 6, 7]. One major advantage of RBM is its universal application to any phase trial and essentially any type of clinical study.

There are, however, barriers to RBM adoption, including challenges in executing RBM within a complex trial workflow (especially when using new technologies or coordinating with multiple stakeholders), concern regarding regulator acceptance of data, a number of country-specific regulatory limitations, sponsor reluctance on certain types of trials, and sponsor sensitivity to inspector findings at the site level. Despite these challenges, RBM is supported and encouraged by multiple regulatory agencies [5, 7, 8]. In fact, these authorities encouraged increased use of RBM as the COVID-19 pandemic unfolded, with travel restrictions, risk of infection for vulnerable patients, and site closures disrupting all aspects of clinical trials, including regular on-site monitoring activities [8, 9].

To shed light on the state of RBM adoption and implementation, the Association of Clinical Research Organizations (ACRO)—a trade association of CROs and technology companies—conducted a landscape survey of RBM use in clinical trials ongoing at the end of 2019 that were managed by several of its member companies. After COVID-19 emerged as a worldwide threat in early 2020, ACRO then gathered additional data from January–June 2020 to determine the impact of the pandemic on trial management, with a specific focus on monitoring. Here, we present both datasets, discussing the insights gained from them into the past and present use of RBM and how changes in trial practices during the pandemic could help shape the future of clinical trial monitoring.

## Methods

### RBM Landscape Survey

Seven ACRO member companies responded to a survey of RBM practices in clinical trials where project management and/or clinical monitoring were within scope of the companies’ services​. A neutral outside vendor collected, blinded, aggregated, and analyzed the data. The dataset included trials that were ongoing as of December 31, 2019, including studies initiated in 2019 and multi-year studies from years prior.

To better understand the RBM landscape, companies participating in the survey were asked to provide data showing how many of their trials implemented the eight RBM/RBQM components: initial cross-functional risk assessment, ongoing cross-functional risk assessment, QTLs, KRIs, Centralized monitoring, off-site/remote-site monitoring, reduced SDV, and reduced SDR. The component definitions presented above were formulated by the authors to provide a good benchmark to support data collection, ensuring that data submissions were consistent in the survey.

### Assessment of Trial Disruptions During the COVID-19 Pandemic

Data on RBM detection of on-site/remote visits and protocol deviations from January–June 2019 were provided by three member companies. Further data on trial disruptions during the same period were gathered from additional member companies, as noted.

## Results

### RBM Landscape Data

The landscape survey of RBM implementation during 2019 included 6,513 clinical trials managed by 7 of ACRO’s CRO member companies. Of the included trials, 47% had at least 1 of the 8 RBQM components (listed above), while 53% had more traditional trial management.

**Fig. 1:**
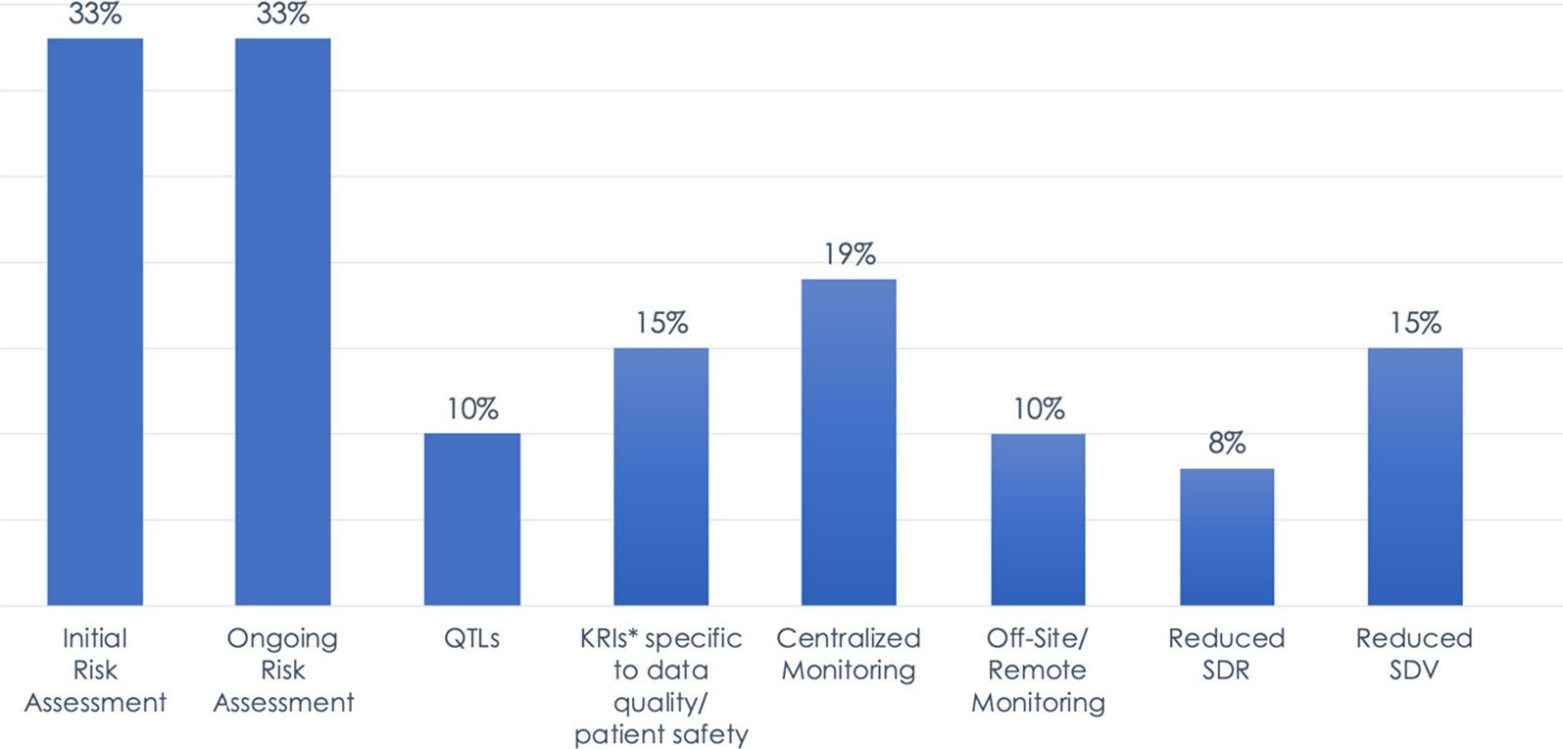
2019 Landscape of RBM/RBQM Components in Clinical Trials. Data represent the percentage of all 6,513 trials included in the survey, not just the subset of studies that have at least one RBM component. *The KRI percentage does not include KRIs related to operations or performance.

Implementation rates for the 5 RBM components (KRIs, centralized monitoring, off-site/remote-site monitoring, reduced SDR, reduced SDV) ranged from 8 to 19% of trials and were markedly lower than the implementation rates for initial or ongoing risk assessments (33% for both), which are specific to RBQM but critical to the execution of RBM (Fig. 1).

**Fig. 2:**
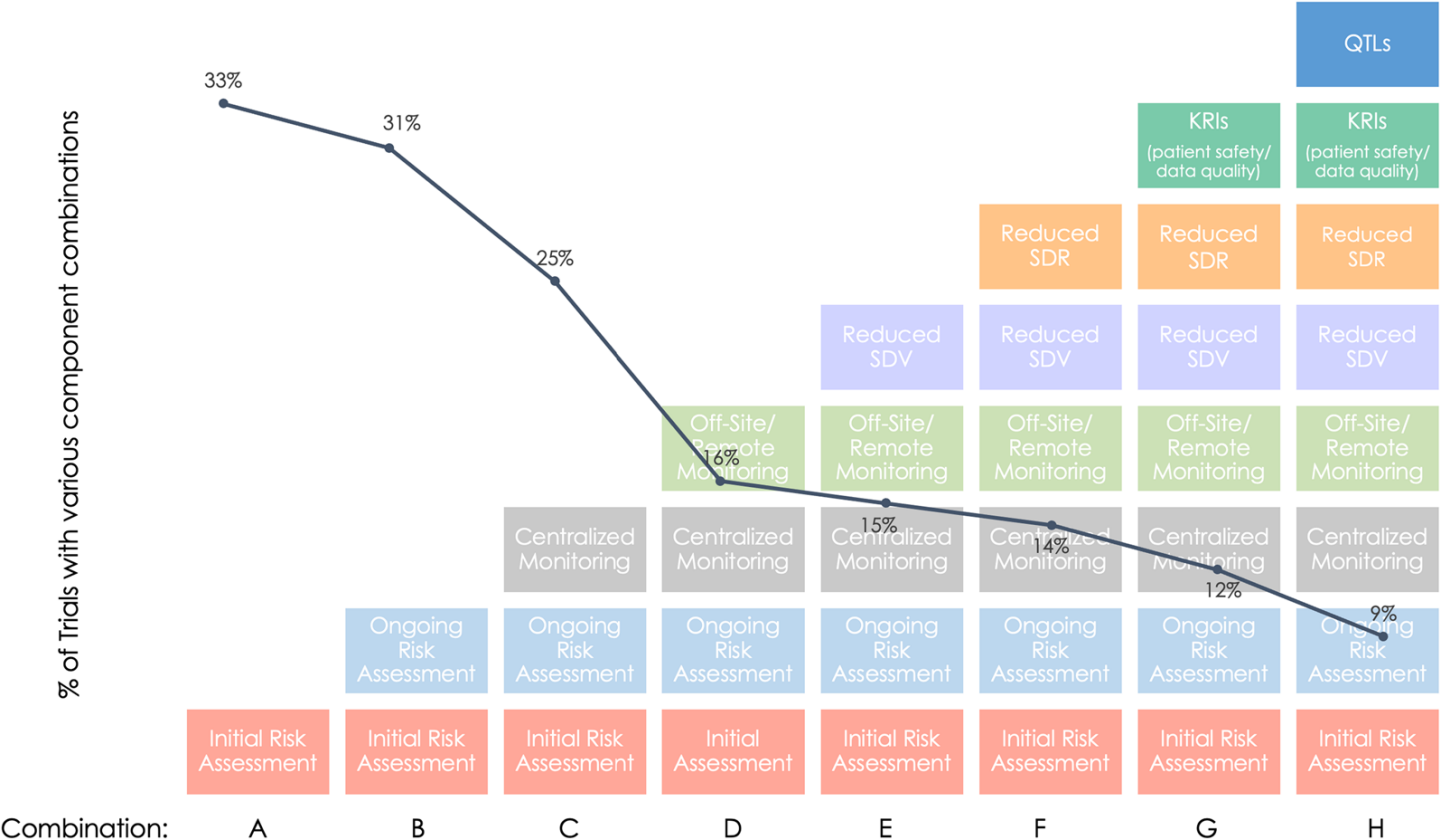
2019 Implementation of RBM/RBQM Components in Combination. Graph shows only some of the common combinations of components and not all combinations reported in the dataset.

Looking at the percentage of trials with specific combinations of RBM/RBQM components (Combinations A–H in Fig. 2), it is clear most trials employed neither a “holistic” RBQM approach (defined as having seven or eight RBM/RBQM components) nor a full-RBM approach (defined as having all five RBM components). Taking as a point of reference trials having both initial and ongoing risk assessments (Combination B), when centralized monitoring is added (Combination C) there is a 6-percentage-point drop in the percentage of total trials and a 9-point drop when off-site/remote-site monitoring is added (Combination D). Adding reduced SDR (Combination E) results in a smaller 1-point drop in the percentage of total trials.

Taken together, the RBM landscape data show that industry adoption is less widespread than expected and implementation is rather piecemeal, with few studies incorporating all five RBM components. These findings also provide a benchmark to better assess future changes in RBM uptake, particularly in situations where trial protocols and regular monitoring practices have been disrupted.

### Impact of the COVID-19 Pandemic on Clinical Trial Monitoring

On March 11, 2020, the World Health Organization (WHO) declared the COVID-19 outbreaks spreading across the globe to be a pandemic. This unprecedented worldwide disruption presented major challenges in clinical trial management by forcing companies to rely mainly on remote and centralized monitoring due to site closures and stay-at-home orders. At the same time, the pandemic created something of a “natural experiment,” allowing ACRO to collect early data on the impact of this shift in trial monitoring to complement the larger-scale RBM landscape dataset.

**Fig. 3:**
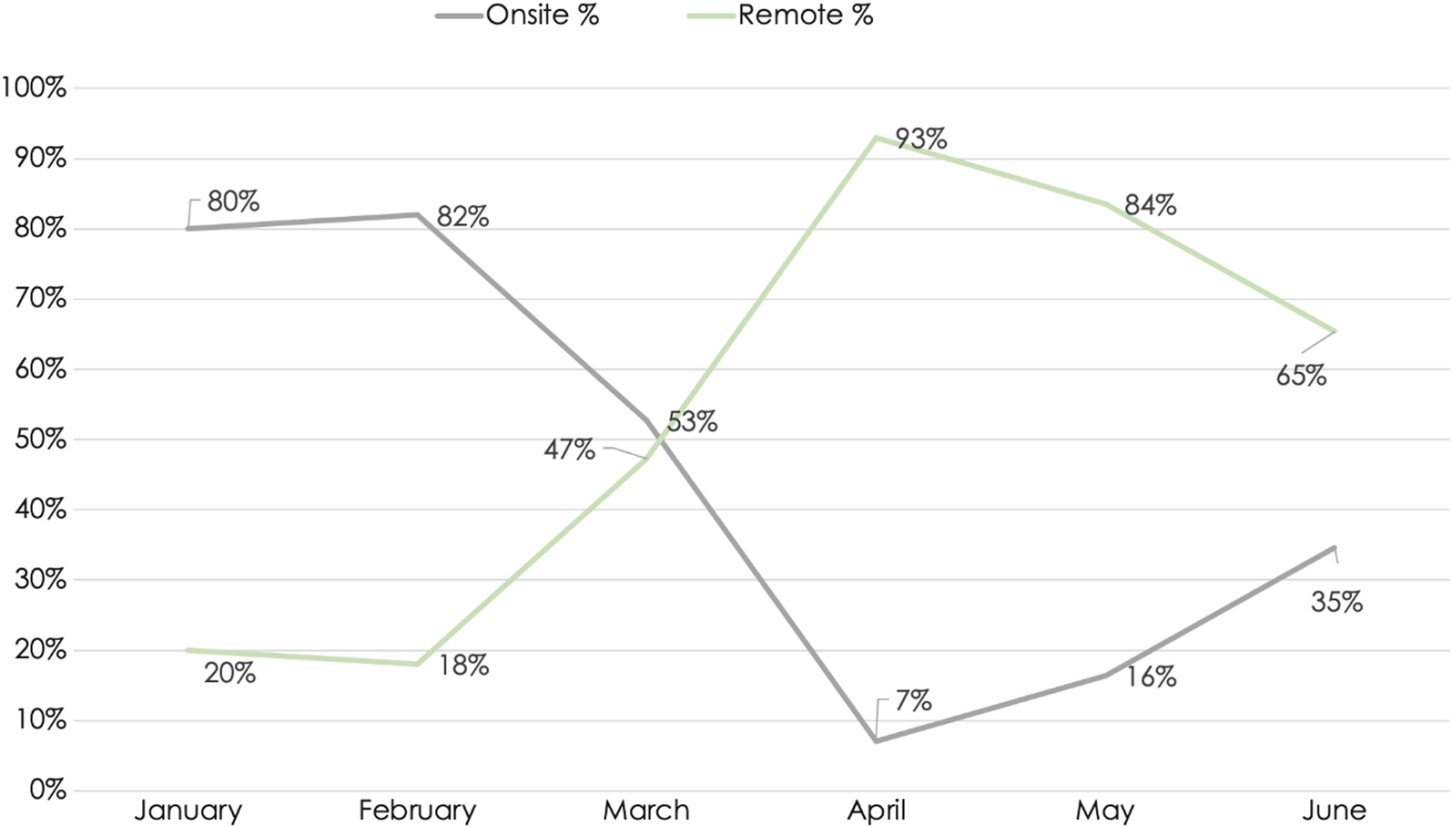
Increased Remote Monitoring Visits and RBM in Response to the COVID-19 Pandemic. Graph shows data from 1 of 3 companies providing monitoring visit data (n =  ~ 1,200 trials), but trends were similar across all 3 companies.

Data from 3 member companies covering trials from January–June 2020 showed remote-site monitoring increased and on-site monitoring decreased at the peak of the pandemic in April compared with the pre-pandemic baseline in February (representative data covering ~ 1,200 trials from 1 company shown in Fig. 3). These trends began to reverse themselves post peak, but the percentage of remote-site monitoring visits was still markedly higher in June compared to the baseline percentage.

**Fig. 4:**
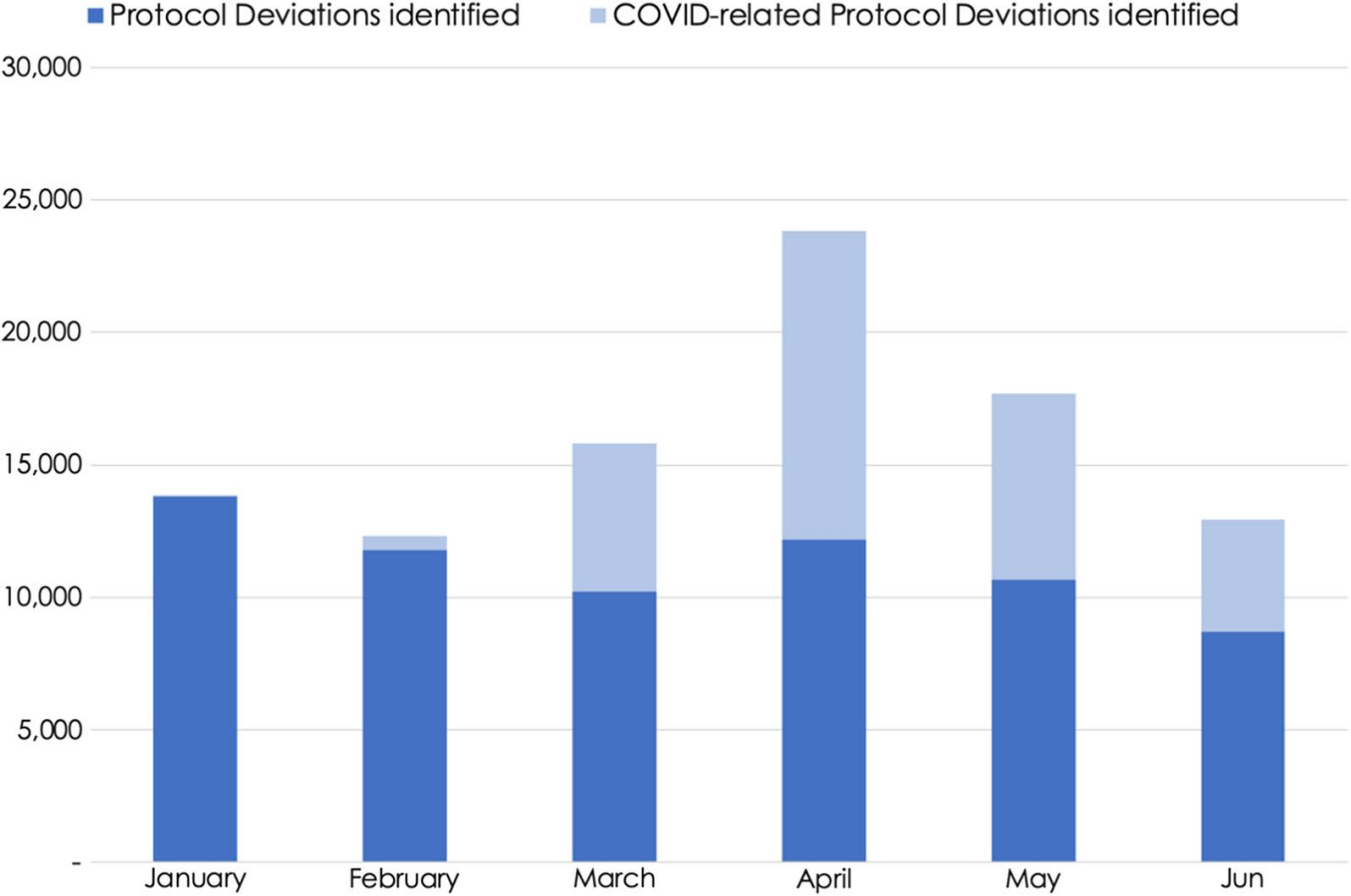
Clinical Trial Protocol Deviations Detected by RBM During the COVID-19 Pandemic. Graph shows data from 1 of 3 companies providing protocol deviation data (n =  ~ 1,200 trials), but trends were similar across all 3 companies.

Remote-site monitoring effectively captured protocol deviations as the pandemic evolved, even during the peak in April 2020 when there was little or no physical access to most trial sites (representative data covering ~ 1,200 trials from 1 company shown in Fig. 4). A corresponding peak in COVID-related protocol deviations was also seen that month, declining over time through June, but remaining above the pre-pandemic levels. Notably, the total non-COVID protocol deviations detected each month from March to May were similar to the February baseline, even as the percentage of remote-site monitoring visits increased from 18% in February to a high of 93% in April. This suggests that the rapid shift in monitoring methods allowed for sufficient oversight and monitoring continuity, lending confidence in data quality and patient safety.

### Trial Disruptions During the COVID-19 Pandemic

Additional data shed more light on the scale of the trial disruptions as the pandemic approached its first peak, complementing the monitoring data. In less than a month (March 14–April 6, 2020), 1 company reported that the percentage of institutions where patient or site monitoring visits for the company’s trials were disrupted jumped from 10 to 49%. A second company reported that 33% of planned trial visits were disrupted in March, and by the end of March, approximately 70% of sites were inaccessible. New subject enrollment in trials managed by a third company was reduced by 65% in March 2020 compared with March 2019.

Though less comprehensive than the pre-COVID RBM implementation data, the mid-pandemic trial monitoring and disruptions data help illustrate the mitigating effect of one RBM component on pervasive and potentially crippling disruptions to clinical trial management.

## Discussion

### The RBM Landscape

The RBM landscape data—generated from a survey planned before the COVID-19 pandemic covering more than 6,000 clinical trials—provide a pre-pandemic baseline for RBM adoption and implementation. The data collected for this analysis are representative of studies where CROs have contracted services. We acknowledge these data may not be reflective of the entire clinical trial development landscape, as CROs may only perform certain activities outsourced from trial sponsors and not others; however, it is our opinion that this dataset still provides valuable visibility into clinical trial implementation of RBM. Overall, execution of RBM is rather piecemeal, with the individual RBM components being used in 8%–19% of trials and very few trials executing a full-RBM approach. This inconsistent implementation is also seen for the RBQM components critical to the success of RBM, with initial and ongoing risk assessments each implemented in less than half of trials and not always together in the same trial. Not surprisingly, given the poor uptake of RBM, use of holistic RBQM is quite rare, meaning that the full potential of RBM to enhance trial quality—by more efficiently detecting errors compromising patient safety and data validity so that their impact can be mitigated—is not yet being realized.

The key takeaway from the landscape data is that industry adoption of RBM is less extensive than expected, likely because companies are reluctant to fully commit to changing their existing practices and protocols. For example, centralized monitoring, the most frequently used RBM component, was implemented in less than 20% of the trials in our dataset; however, off-site/remote-site monitoring was used in only 10% of the trials, suggesting greater acceptance of remote data evaluation than replacement of on-site visits with remote visits.

One reason often cited for the incomplete adoption and partial implementation of RBM is a hesitance on the part of trial sponsors and CROs to reduce the amount of SDR/SDV in favor of a more targeted approach. For example, site inspections or audits that find discrepancies not critical-to-quality may cause study personnel to rely more on SDV/SDR, even though patient safety and data integrity have not been compromised. In our experience, most sponsors who agree to reduced SDV also accept reduced SDR, and our landscape data are generally consistent with this assessment. There is, however, resistance to reducing SDR (i.e., if you do not look at 100% of the source data, how do you ensure that you do not miss any adverse events?), as shown by the lower implementation rate for reduced SDR (8%) compared with reduced SDV (15%). Despite this, we believe that a risk-based approach to SDR/SDV best serves the interest of sites, patients, and the whole of the clinical research community.

Other explanations for slow RBM adoption are lack of familiarity with different RBM practices, misconceptions that it might not fit into all studies, the complexity of implementing these practices, logistical barriers, the need for new and unfamiliar technology, and an incorrect assumption that RBM methodology data are less likely to satisfy regulators. Many of these challenges can be addressed by educating study sponsors and personnel on RBM implementation and the regulatory landscape, and also managing expectations regarding what efficient monitoring that meets regulatory guidance looks like. At the same time, stronger, more specific regulator guidance and alignment within regulatory agencies is needed, particularly when executing RBM as part of a more holistic end-to-end RBQM framework.

### Real-World RBM Implementation

As disruptive as the COVID-19 pandemic has been, it also created a natural experiment by motivating many companies to transition to remote-site monitoring over the same time period to avoid trial interruptions. The real-world utility of a single RBM component—remote-site monitoring—is thus shown in the mid-pandemic data, which offer an early snapshot of adaptations in trial practices, showing that COVID-related protocol deviations were successfully differentiated from non-COVID-related deviations and that little change in the monthly totals of non-COVID deviations was seen even in the absence of physical access to sites. Early data suggest that the effectiveness of remote-site monitoring mid-pandemic was similar to that of on-site monitoring pre-COVID; however, without established RBM principals and recent technological advancements, there likely would have been a larger disruption in monitoring that would have impeded the continuation of many trials.

Based on the present data and a wealth of experience in RBM, as experts in the field, we recommend the following considerations for changes in monitoring methodology due to trial disruptions:

- Centralized monitoring and risk-based approaches help provide confidence in the quality of data monitored during external disruptions.
- While alternative ways of accessing source documents may be warranted for certain purposes, such as safety oversight or critical endpoint collection in pivotal trials, achieving 100% SDR/SDV for interim analysis or database lock is *not* an exigent circumstance that warrants unplanned-for remote access to source documents.
- Regulatory consistency and direction are needed for remote-site monitoring and resumption of monitoring activities as restrictions are relaxed, with a focus on the benefits of monitoring critical data only.
  o On April 16, 2020 the FDA clarified its thinking in regard to remote access, emphasizing that sponsors should carefully evaluate technologies and take a risk-based approach to the unplanned-for collection of source data from sites and document changes to the trial monitoring plan.
  o While we appreciate FDA mentioning risk-based approaches, we think there is potential for stakeholders to interpret the FDA’s flexibility on remote SDV practices as implicit endorsement of 100% SDV now and going forward.

## Conclusion

The RBM landscape survey showed that RBM adoption before the COVID-19 pandemic was not as widespread as expected, despite the proven benefits and clear potential of this approach. In addition, few trials implement more than a few of the eight RBM/RBQM components, meaning the full potential of RBM as a vital part of a broader trial management framework is far from being realized. What is clear from the rapid shift from on-site to remote-site monitoring for most clinical trials during the pandemic is that transitioning to an RBM approach without diminishing monitoring effectiveness is possible, even in difficult circumstances.

The current findings and a wealth of practical experience support the uptake of RBM and, potentially, a shift to RBQM. We believe the industry will continue to lean into greater adoption of off-site/remote-site monitoring and other RBM practices in a post-pandemic environment. To facilitate this, companies involved in clinical trial research are encouraged to share their real-world experiences with RBM implementation pre- and mid-pandemic, both the successes and the lessons learned. ACRO will continue gathering data on trial monitoring practices through the pandemic and after it has ended, with the aim of sharing our findings with the larger clinical research community. We further encourage the industry at large to continue to advance best practices and promote adoption of RBM.
